# Supplementary material for: Patterns of Intron Gain and Loss in Fungi
Source: PLoS Biol. 2004 Nov 30;2(12):e422. doi: 10.1371/journal.pbio.0020422 (PMC532390; doi:10.1371/journal.pbio.0020422)
Supplement: Table S1 — Also available at http://genes.mit.edu/NielsenEtAl/. (4.3 MB ZIP). [file pbio.0020422.st001.zip › NielsenEtAl/html/903.html]

AN6902.1.NCU07547.1.MG08908.1.FG06705.1


```
 CLUSTAL W (1.82) Multiple Sequence Alignments - Introns Inserted


Sequence 1: MG08908.1	251 aa
Sequence 2: FG06705.1	244 aa
Sequence 3: NCU07547.1	252 aa
Sequence 4: AN6902.1	246 aa
Alignment Length: 255 aa
Number Identitical Residues: 103 aa
Alignment Score (without introns) 5672


MG08908.1 	MPKSKRAKVVHMTNVAKK-TREQKELLFTNIRECVPQYQHLFVFSVDNMRNNNLKDVRRE
NCU07547.1	MPKSKRAKVYNLTQVTKK-NREQKEKLFENIRECIPNYQHCFVFSIDNMRNNYLKDVRKE
FG06705.1 	MPKSRRAKVVHLTQVDKK-TRENKDKLFQNIRDTVPEYQNCFVFSVDNMRNNHLKDVRRE
AN6902.1  	MPRSKRARIVHESKTAKKSHKEQTRRLYANIRECVEKYDHLFVFSVDNMRNTYLKDVRTE
          	**:*:**:: : ::. **: :*:.  *: ***: : :*:: ****:*****. ***** *

MG08908.1 	LSGDSR2MFFGKTKLMARALGQTAEDEQAEGLHKLTSHLAGTVGLIFTNRPASELLSYLD
NCU07547.1	LN-DCR2IFFGKTKLTARALGTTPEDAQADGLDKLSKYLSGSVGLIFTNRDPSEIKDYFV
FG06705.1 	LS-DCR2VFFGKTKLMAKALGQTPEEAIAPGIEHLSKYFTGTVGLILTNRPAEEILTYFE
AN6902.1  	FA-DSR2LFFGKTKVMAVALGHNPETEAAENLHLLTPYLTGAVGLLFTSRDPASVTDYFD
          	:  *.* :******: * *** ..*   * .:. *: :::*:***::*.* . .:  *: 

MG08908.1 	SITSVDFARAGAVAPRDFSIPPGVVYSTGGEVPQEYDVPLAHSIEPELRKLGVPTRLIKG
NCU07547.1	NLTQVDFARAGSVATRTITIPSGPLYSTGGEVPAEHDVPVSHTLEPELRRLGMPTRMVKG
FG06705.1 	NLAPVDFARAGAVATRDFSIPTGVVYATAGEVPAEHDVPLAHTIEPELRRLGVPTRMVKG
AN6902.1  	AFRPLDFARAGTEATRSFSIPAGLVYSRAGEIPTSEDEPVSHTIEPELRKLGVPTRLVKG
          	 :  :******: *.* ::**.* :*: .**:* . * *::*::*****:**:***::**

MG08908.1 	RVVLGGENGEGSTGYDVCREGDVLDSRQTRLLKLFSVCFSEFKIQVLA2YWSAATGEVTE
NCU07547.1	KVCLGDEAGEGDD-YVICKEGETLDSRQTRLLKLFSICLSEFRVKLLA~YWSAASGEVTE
FG06705.1 	RVVLGDESGQGEE-YTVCKEGDVLDSRQTRLLKLFDMCMSEFKVKVLA2YWNAGSSEVTE
AN6902.1  	KVML--ELTDGQEGYPVCKKGEILDSRQTTLLKMFGVQTSEFRVGLKA~HWARATGKVEI
          	:* *  *  :*. .* :*::*: ****** ***:*.:  ***:: : * :*  .:.:*  

MG08908.1 	VNP--GSAGDAIDDSD-
NCU07547.1	LEKPGEAGAEEMEEDDE
FG06705.1 	VN------TNAMDEN--
AN6902.1  	LEK----NENEMEVE--
          	::       : :: .
```
